# Impact of MICA 3′UTR allelic variability on miRNA binding prediction, a bioinformatic approach

**DOI:** 10.3389/fgene.2023.1273296

**Published:** 2023-12-07

**Authors:** Karen Toledo-Stuardo, Carolina H. Ribeiro, Ivo Campos, Samantha Tello, Yesenia Latorre, Claudia Altamirano, Karen Dubois-Camacho, Maria Carmen Molina

**Affiliations:** ^1^ Faculty of Medicine, Immunology Program, Institute of Biomedical Sciences (ICBM), Universidad de Chile, Santiago, Chile; ^2^ School of Biochemical Engineering, Pontificia Universidad Católica de Valparaíso, Valparaíso, Chile; ^3^ Faculty of Medicine, Clinical and Molecular Pharmacology Program, Institute of Biomedical Sciences (ICBM), Universidad de Chile, Santiago, Chile; ^4^ Gastroenterology and Hepatology Department, University Medical Center Groningen, Groningen, Netherlands

**Keywords:** MICA, SNP, miRNA, post-transcriptional regulation, allelic variation, 3′UTR

## Abstract

MicroRNAs (miRNAs) are small non-coding RNAs that participate as powerful genetic regulators. MiRNAs can interfere with cellular processes by interacting with a broad spectrum of target genes under physiological and pathological states, including cancer development and progression. Major histocompatibility complex major histocompatibility complex class I-related chain A (MICA) belongs to a family of proteins that bind the natural-killer group 2, member D (NKG2D) receptor on Natural Killer cells and other cytotoxic lymphocytes. MICA plays a crucial role in the host’s innate immune response to several disease settings, including cancer. *MICA* harbors various single nucleotide polymorphisms (SNPs) located in its 3′-untranslated region (3′UTR), a characteristic that increases the complexity of MICA regulation, favoring its post-transcriptional modulation by miRNAs under physiological and pathological conditions. Here, we conducted an in-depth analysis of *MICA* 3′UTR sequences according to each *MICA* allele described to date using NCBI database. We also systematically evaluated interactions between miRNAs and their putative targets on *MICA* 3′UTR containing SNPs using *in silico* analysis. Our *in silico* results showed that *MICA* SNPs rs9266829, rs 1880, and rs9266825, located in the target sequence of miRNAs hsa-miR-106a-5p, hsa-miR-17-5p, hsa-miR-20a-5p, hsa-miR-20b-5p, hsa-miR-93, hsa-miR-1207.5p, and hsa-miR-711 could modify the binding free energy between −8.62 and −18.14 kcal/mol, which may affect the regulation of MICA expression. We believe that our results may provide a starting point for further exploration of miRNA regulatory effects depending on MICA allelic variability; they may also be a guide to conduct miRNA *in silico* analysis for other highly polymorphic genes.

## 1 Introduction

MicroRNAs (miRNAs) are small non-coding RNAs containing 20–22 nucleotides (nts) that can significantly change gene expression through post-transcriptional regulation by driving target mRNA deadenylation, degradation and translational repression (Huntzinger and Izaurralde, 2011; Shah and Shah, 2020). MiRNAs can affect the expression of one or multiple genes under physiological and pathological states, and dysregulation mechanisms affecting miRNAs can potentially alter biological pathways involved in cancer development and progression. In mammalians, the biogenesis of miRNAs is a highly conserved process that involves the action of type II and III polymerases (Borchert et al., 2006), resulting in the transcription, from a DNA sequence, of a single strand of primary miRNA (pri-miRNA) (∼1,000 nts). Pri-miRNA is processed by a complex formed by the RNA-binding protein DiGeorge Syndrome Critical Region 8 (DGCR8) and a ribonuclease III enzyme, DROSHA, giving rise to a pre-miRNA transcript (∼60 nts), which is further processed by the RNAse III endonuclease Dicer, resulting in a mature miRNA duplex. Argonaute 2 (AGO2), a protein with catalytic activity, associates with miRNA, and this complex generates the miRNA-induced silencing complex (miRISC), which drives the interactions between the miRNAs and their messenger RNAs (mRNAs) targets (OBrien et al., 2018). This interaction depends on the particular configuration between miRNA:mRNA complementarity, which includes the characteristic annealing in the “seed sequence region” (six to eight base pairs from the miRNA 5‘end), and also non-canonical pairing (including nucleotides in miRNA 3′end) conferring stability to the RNA interaction and promoting the effect of miRISC (Brennecke et al., 2005; Wang, 2014). These recognition patterns can be damped by slight changes in the target nucleotide sequence, such as DNA single nucleotide polymorphisms (SNPs) associated with the 3′untranslated region (3′UTR) of a given mRNA, and potentially modify the effect of functional miRNA (Cai et al., 2009). Thus, SNPs in the 3′UTR may abolish or create novel miRNA binding sites (Manikandan and Munirajan, 2014), and polymorphic genes could be more prone to this type of plasticity.

The major histocompatibility complex (MHC) class I-related chain A (MICA) is a stress-induced ligand that plays an important role in the immune response to tumors. It binds to natural-killer group 2, member D (NKG2D) receptor on Natural Killer (NK) cells and other cytotoxic lymphocytes, which triggers the activation of effector cell cytolytic activity and becomes the signal for interferon-gamma (IFN-y) production, thus activating the host immune response mainly against intracellular pathogens and tumor cells (Mah and Cooper, 2016; Fuertes et al., 2021)*. MICA* is a highly polymorphic gene located in chromosome 6 at 6p21.3; it contains six exons: exon 1 encodes the leader peptide, exons 2-4 encode the three extracellular domains α1, α2, and α3, respectively, exon 5 encodes the transmembrane region, and exon 6 encodes the hydrophobic carboxy-terminal cytoplasmic tail or it can be part of 3′UTR in the case of MICA allele * 008 (Vitiani et al., 1998; Li et al., 1999). Currently, more than two hundred *MICA* full-sequences have been described, according to the IPD-IMGT/HLA Database (IPD-IMGT/HLA, 2023). MICA*008 is the most prevalent allele in the human population, followed by MICA*002; together, they comprise almost 50% of the *MICA* alleles described to date (The Allele Frequency Net Database, 2022). Some of them have been associated with increased risks of developing cancer, including gastric (MICA*009/049) (Toledo-Stuardo et al., 2021), and colorectal cancer (MICA*012:01) (Ding et al., 2020). Therefore, *MICA* allelic distribution may contribute to disease risks in specific human populations (Lucas et al., 2008; Wenda et al., 2013; Wang et al., 2016; Yamakawa et al., 2017; Klussmeier et al., 2020).

MiRNAs that have a direct effect on the *MICA* transcript have been associated with degradation and reduction of MICA expression on the tumor cell membrane, which may affect the cytotoxic response mediated by NK cells (Xie et al., 2014). Hence, miRNAs address a fine regulation of transcript, regulating MICA levels mainly in stress conditions (Stern-Ginossar et al., 2008). Post-transcriptional regulation of *MICA* by miRNAs has been reported in different types of cancer and clinically associated with relevant tumor characteristics and disease progression. Some of these miRNAs include hsa-miR-25, hsa-miR-93, hsa-miR-106a, hsa-miR-106b and hsa-miR-373, which were detected in hepatocellular carcinoma cell lines (Kishikawa et al., 2013; Wu et al., 2014; Yang et al., 2015); hsa-miR-146b-5p, which has been related with primary tumor-derived papillary thyroid carcinoma cells (Al-Abdallah et al., 2020); hsa-miR-519a-3p, detected in breast cancer (Breunig et al., 2017), hsa-miR-302c and hsa-miR-520c, present in multiple cancer cell lines (Kasumi-1, K-562, MDA-MB-231, MCF-7, and HEK-293T) (Min et al., 2013). In contrast, a miRNA that upregulates MICA expression has also been reported, such as miR-125b, whose upregulation leads to increased MICA levels in human myeloma cells, affecting the immune response against tumors (Abruzzese et al., 2016). The presence of SNPs located in the 3′UTR region of the *MICA* gene with very strong linkage disequilibrium (LD) allowed the classification of the MICA 3′UTR into seven types, UTR1-7 (Luo et al., 2013). These SNPs can affect the strength of miRNAs interaction. *MICA* gene harbors more than 300 SNPs (MICA ENSG00000204520, variant table - Ensembl release 109 (Gene: MICA ENSG00000233051, 2023; Martin et al., 2023)) that may permit a gain or loss of allele-dependent miRNA target sequence recognition, which gives plasticity to miRNA regulation mechanisms, thus affecting protein expression. Such versatility of miRNA targets conferred by genetic variants of *MICA* gene has not been completely approached.

Here, we studied miRNAs with potential novel binding sites to SNPs located in *MICA* 3′UTR by *in silico* analysis; we also discuss their possible implications in the regulation of MICA expression in cancer, emphasizing the relevance of *MICA* alleles in this post-transcriptional modulation. We hypothesize that the miRNAs evaluated herein could determine MICA expression depending on the allele. We believe our data contribute to the knowledge of how *MICA* alleles variability can influence miRNA binding, and present rational *in silico* evidence for future MICA expression studies.

## 2 Methods

### 2.1 *MICA* 3′UTRs identification and analysis

The most frequent *MICA* alleles worldwide were selected for analysis: MICA*001, *002, *004, *007, *008, *009, *010, *011, *012, *015, *017, *018, *019, *027, *049, *068 (http://www.allelefrequencies.net). NCBI reference sequence used for analysis was NM_001177519.3. The *MICA* 3′UTR mRNA polymorphic sequences were reviewed on IPD-IMGT/HLA Database, and the initial sequence and length of 3′ UTR regions were analyzed by using the IMGTplatform (IPD-IMGT/HLA, 2023; Lefranc et al., 2003)).

#### 2.1.1 Classification of *MICA* 3′UTR types

In previous studies, the classification of *MICA* 3′UTR types was based on the presence of 9 SNPs; rs9266826 (G/A), rs9266827 (A/G), rs9266828 (G/C), rs9266929 (A/G), rs140390705 (C/T), rs 1880 (C/A), rs113015830 (A/T), rs1131904 (A/G), and rs9266831 (G/A) were grouped into UTR1-UTR7 (Luo et al., 2013). However, in the present study, we introduced a new 3′UTR classification that considers: 1) the introduction of two SNPs, rs9266825(C/A) and rs 1882 (A/G), as they are located in regions with LD with the other 9 SNPs described (from position 1,000 to 1,327 (NM_001177519.3)); 2) exclusion of UTR5 and UTR6, which harbor the SNPs rs140390705 allele T and rs113015830 allele A, respectively, as they have a lower global frequency (0.0006 and 0.0044, respectively), and they have not been identified as part of *MICA* alleles reported in the IPD-IMGT/HLA Database (Table 1); 3) in consideration of a guanine insertion in the transmembrane region at position 952 located in exon 5 (*MICA* *008), and a deletion of guanine at position 892 in exon 4 (*MICA* *015, and *017), both of which change the reading frame, resulting in premature stop codons TAA or TGA at positions +997 and +1,035, respectively. The rest of the alleles maintain TAG stop codon at position +1,154. Thus, MICA*008 3′UTR is longer than the 3′UTR from *MICA* *015, and *017 MICA alleles and the other MICA alleles described to date. *MICA* alleles reported in the IPD-IMGT/HLA Database are included in Table 1.

**TABLE 1:** Diversity of 3′UTR of the MICA gene and novel classification.

Single nucleotide polymorphisms (SNPs)/NM_0011775193 position	Global allele frequency (1,000 genomes)	UTR1a	UTR1b	UTR1c	UTR2a	UTR2b	UTR2c	UTR3	UTR4	UTR5	UTR6	UTR7
**rs9266826/1,210**	G = 0.6571	G	G	G	A	A	A	DEL	A	A	G	G
	A = 0.3429											
**rs9266827/1,216**	A = 0.6571	A	A	A	G	G	G	DEL	G	G	A	A
	G = 0.2429											
**rs9266828/1,220**	G = 0.6571	G	G	G	C	C	C	DEL	C	C	G	G
	A = 0.3429											
**rs9266829/1,242**	A = 0.6418	A	A	A	G	G	G	DEL	G	G	A	G
	G = 0.3582											
^**(#)**^ **rs140390705/1,265**	C = 0.9994	C	C	C	C	C	C	DEL	C	T	C	C
	T = 0.0006											
**rs 1880/1,272**	C = 0.7736	C	C	C	A	A	A	DEL	C	A	C	C
	A = 0.2764											
^**(#)**^ **rs113015830/1,311**	T = 0.9956	T	T	T	T	T	T	DEL	T	T	A	T
	A = 0.0044											
**rs1131904/1,349**	A = 0.6573	A	A	A	G	G	G	DEL	G	G	A	A
	G = 0.3427											
**rs9266831/1,361**	G = 0.6571	G	G	G	A	A	A	DEL	A	A	G	G
	A = 0.3429											
**rs9266825/1,122**	C = 0.6571	C	C	C	A	A	A	DEL	A	A	C	C
	A = 0.3429											
**rs 1882/1,151**	A = 0.2768	A	G	G	G	G	G	DEL	G	G	G	A
	G = 0.7232											
**MICA Alleles (p.e)**		*008	*004	*010	*002	*007	*015	NA	*001	NA	NA	*011
			*009	*019			*017		*012			
			*049	*027					*018			
			*068									

(#): SNPs, with a global frequency lower than 0.005. These SNPs, were excluded for our analysis, resulting in the exclusion of UTR5 and UTR6, which are considered analogous to UTR2 and UTR1 respectively. NA: There are no MICA, alleles reported in the HLA, database.

### 2.2 Recognition of miRNAs binding sites to SNPs located in *MICA* 3′UTR

To estimate the miRNAs with potential binding sites to different *MICA* 3′UTR alleles, we performed a primary search for miRNAs with described binding capacity to all *MICA* 3′UTRs, according to the SNPs harbored in this region, using miRbase search tool (https://www.mirbase.org/). We also used the miRBD target prediction database to search for additional miRNAs with targets on SNP-containing *MICA* 3′UTR (Chen and Wang, 2020).

### 2.3 Bioinformatic analysis of energy and type of interaction between miRNAs and *MICA* 3′UTR

**FIGURE 1:**
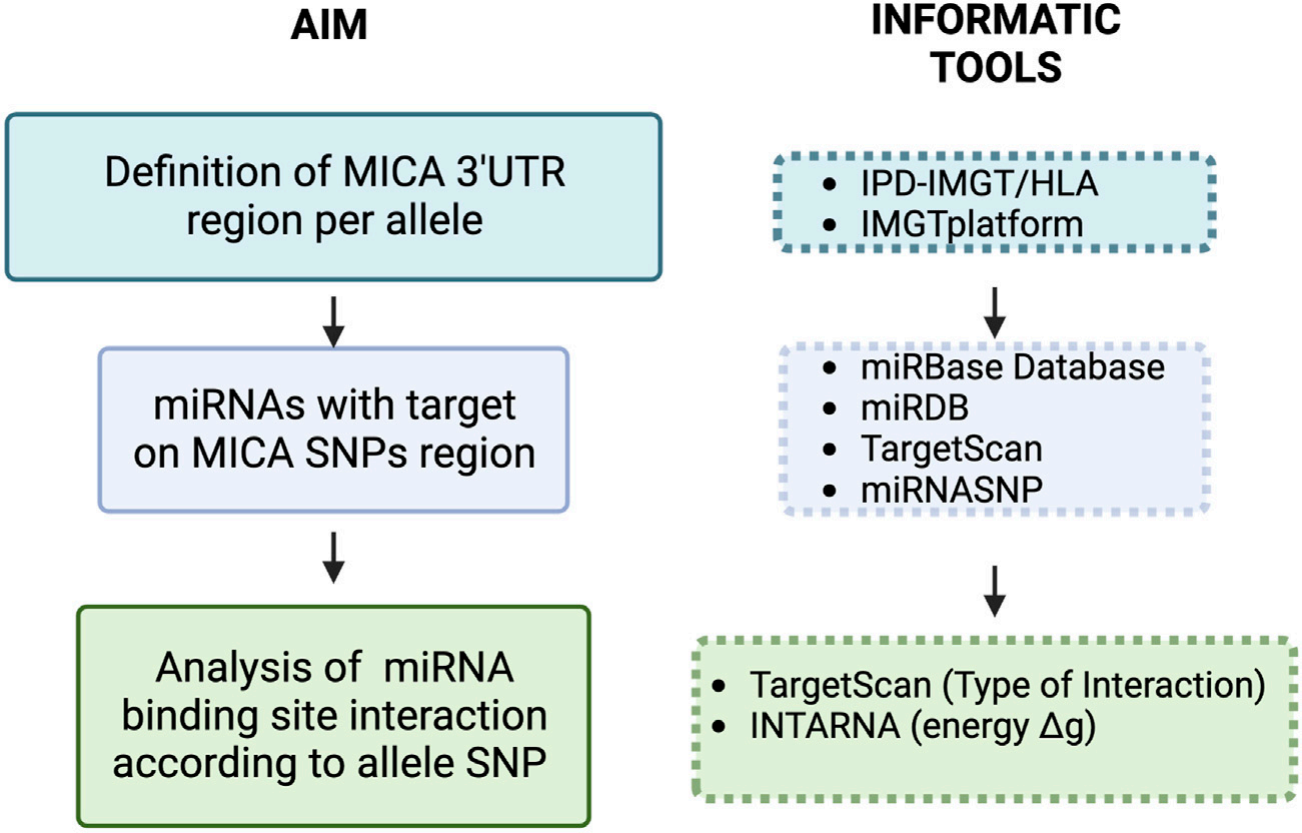
Workflow of the methodological approach for target prediction of miRNAs with interaction sites in SNPs located in the 3′UTR of MICA. Initially, we defined the 3′UTR of MICA alleles to further search for miRNA target sequences containing SNPs. MICA transcript nucleotide sequence NM_001177519.3 was used for this purpose, and the different 3′UTRs were analyzed using the mirbase miRNA search tools [39–41] (Table 1). We also used the miRBD target prediction database to look for additional miRNAs with targets on SNPs in MICA 3′UTR [42]. Next, we analyzed the strength of the ΔG between the selected miRNAs and their targets; such analysis was carried out using IntaRNA web server [43–45]. We selected those miRNAs with the highest probability of effect (minimal energy profile - ΔG: < −8 kcal/mol); the type of canonical miRNA interaction (8-mer, 7mer-A1, 7mer-m8) was then analyzed using TargetScan [46]. The miRNAs with MICA targets that have been previously reported were further analyzed according to their target sequence with SNPs in MICA, the ΔG (IntaRNA), and the type of miRNA interaction (TargetScan).

The strength of the interaction between the selected miRNAs and their target sequence (*Gibbs free energy*: ΔG) was analyzed using IntaRNA web server (Bernhart et al., 2008; Chitsaz et al., 2009; Mann et al., 2017). The minimal free energy (MFE) for miRNA selection, to cover possible miRNA::RNA interactions, was lower than −8.00 kcal/mol. Only miRNAs interaction with canonical seed sequence features, according to TargetScan, was considered (8-mer, 7mer-A1, 7mer-m8) (Agarwal et al., 2015). We also analyzed previously reported miRNAs with *MICA* 3′UTR binding sequences not containing SNPs using the same search tools. The general strategy for miRNA search and data analysis is illustrated in Figure 1.

### 2.4 Evaluation of the impact of SNPs on miRNA target sites

To evaluate the impact of SNPs in *MICA* alleles on miRNA interaction, we used the difference of ΔG between SNP alleles, as previously reported by Manikandan & Kannan Munirajan (Manikandan and Munirajan, 2014).

$$\Delta \text{MFE}=\left(\text{MFE of miRNA}[{\text{mRNA duplex}}^{\text{ancestral allele}}]\;\right)\;‐\;\left(\text{MFE of miRNA}[{\text{mRNA duplex}}^{\text{derived allele}}]\;\right)$$

In addition, to complement the identification of gain/loss of miRNA binding sites by SNPs, we used the bioinformatic tool miRNASNP (Liu et al., 2021).

### 2.5 Folding of 3′UTR secondary structure analysis

To predict the probability of 3′UTR RNA folding, as well as its thermodynamic parameters, we used the RNAfold WebServer from ViennaRNA WebServices (Lorenz et al., 2011) and the UNAfold tool, respectively.

### 2.6 Phylogenetic tree analysis of the MICA 3′UTR types

Coding DNA HLA sequences from IPD-IMGT/HLA (https://www.ebi.ac.uk/ipd/imgt/hla/) were aligned using Clustal Omega EMBL-EBI Webserver applying the *Neighbour-joining tree* without distance corrections analysis (Madeira et al., 2022).

## 3 Results

### 3.1 Analysis of MICA 3′UTR according to alleles and SNPs

**FIGURE 2:**
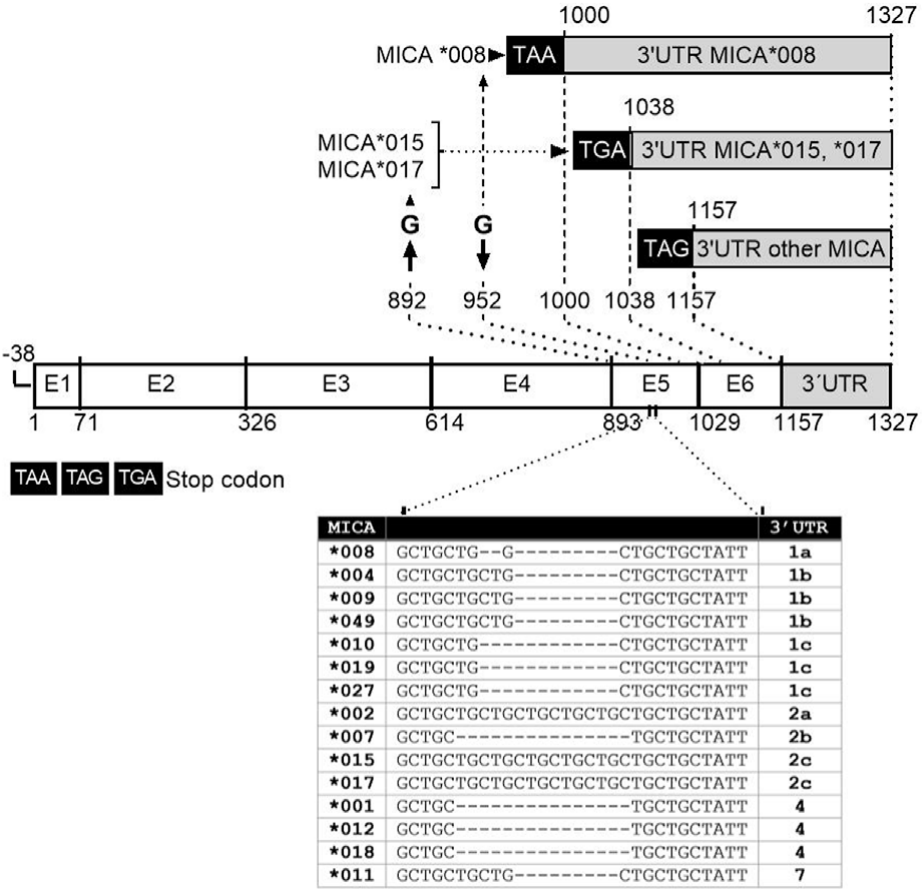
Schematic representation of the gene structure of main MICA alleles studied. MICA gene is composed of six exons; the length of MICA alleles sequences depends on deletions or insertion in exon 5. The MICA∗008 allele includes a guanine insertion in the transmembrane region at position 952 located in exon 5. The MICA *015 and *017 alleles lack guanine at position 892 in exon 4. These modifications change the reading frame, resulting in the generation of premature stop codons TAA or TGA at position +997 and +1,035 of these alleles, respectively. The rest of the alleles maintain TAG stop codon at position +1,154. MICA*008 3′UTR is longer than the 3′UTR from alleles *015 and *017, as well as the rest of MICA alleles analyzed. MICA alleles showed variations in the number of repetitions of the GCT triplet within exon 5, known as short tandem repetitions (STRs) (Table inserted). These triplets encode alanine in the protein, leading to different lengths of this region across MICA alleles.

**FIGURE 3:**
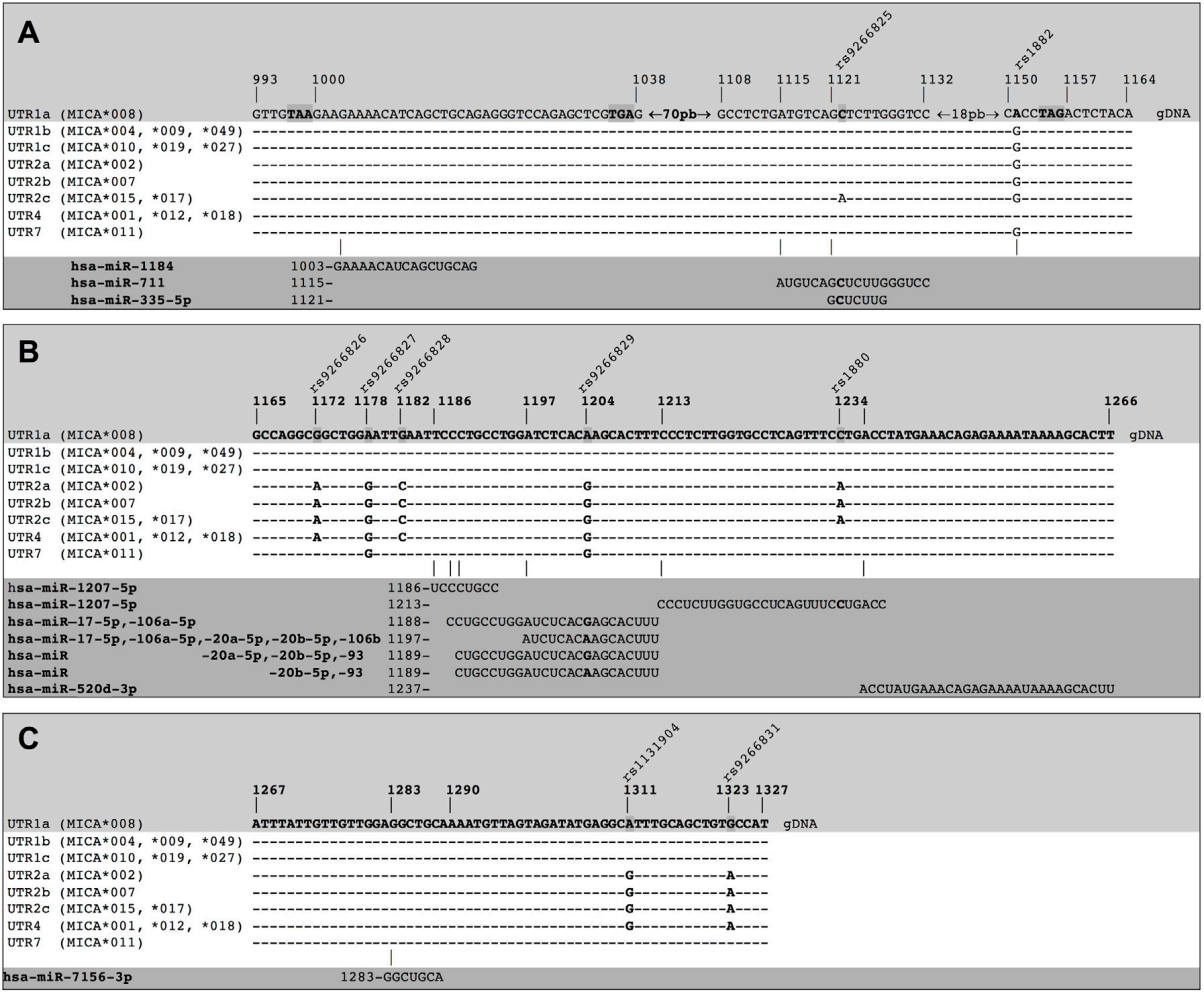
Binding sites of miRNA on different MICA 3′UTRs. The different types of 3′UTRs of MICA alleles show a combination of nine polymorphisms (highlighted in the light grey zone) and aligned in each case (white zone). The presence of polymorphisms leads to differences in the binding of miRNAs. The miRNAs and the binding sites sequences in the MICA 3′UTRs are also detailed (dark grey zone). **(A)** Hsa-miR-1184 shows a preferential binding site within UTR1a, as this region is a coding sequence (CDS) in the other alleles. Furthermore, hsa-miR-711 and hsa-miR335-5p have a binding site preferentially within UTR1a and UTR2c. **(B)** hsa-miR-1207-5p shows two different binding sites. Hsa-miR-17-5p, hsa-miR106a-5p, hsa-miR-20a-5p, hsa-miR-20b-5p, hsa-miR-106b, and hsa-miR-93 also show multiple binding sites that involve polymorphisms (bold nucleotide). The hsa-miR-520d-3p shows a binding site to a non-polymorphic region. **(C)** hsa-miR-7156-3p also shows a binding site to a non-polymorphic region, binding to all types of MICA 3′UTRs. gDNA: genomic sequence of DNA.

To gain a comprehensive understanding of the 3′UTR regions of the most frequent *MICA* alleles, we conducted an analysis to define the specific characteristics of each allele, taking into account the presence of stop codons and SNPs. The MICA*015 and *017 alleles lack guanine at position 892, the last nt in exon 4. These changes in the genetic sequence result in a modification of the reading frame, leading to the generation of premature stop codons: TAA (position +997) for MICA*015 and TGA (position +1,035) for MICA*017 (Wang et al., 2016) (Figure 2, 3). The rest of the alleles maintain TAG stop codon (position +1,154). In turn, the MICA*008 allele includes a guanine insertion in the transmembrane region at position 952 located in exon 5. Thus, MICA*008 3′UTR harbors a longer sequence, with 327 nts (position +1,000 to +1,327), compared to the 3′UTR from alleles *015 and *017, which present 289 nts (position +1,038 to +1,327); the remaining MICA alleles contained 170 nts (position +1,157 to +1,327). *MICA* 3′UTR of *015 and *017 alleles, as well as the remaining *MICA* 3′UTR sequences, comprised nucleotides from positions 1,038 to 1,327 and 1,157 to 1,327, respectively, using as reference the NCBI Reference Sequence NM_001177519.3. Figure 2 summarizes the lengths of the 3′UTR sequences according to *MICA* alleles.

Considering the variations in the *MICA* 3′UTR sequences, including differences in the length and the presence of SNPs, we adopted a novel categorization system. We subdivided the 3′UTRs into distinct categories: UTR1a (*008), UTR1b (*004, *009, *049), UTR1c (*010, *019, *027), UTR2a (*002), UTR2b (*007), UTR2c (*015, *017, *068), UTR4 (*001, *012, *018), and UTR7 (*011). UTR3, UTR5, and UTR6, as classified by Luo and cols (Luo et al., 2013), were excluded from our analysis due to the presence of a deletion (in UTR3) or because of their low global allele frequencies for the SNPs rs140390705 (for UTR5) and rs113015830 (for UTR6)**.** The new classification aligns with the clade grouping in the phylogenetic tree analysis (Supplementary Figure S1) based on sequence similarity. This novel UTR classification reflects that the UTR2a, UTR2b, UTR4, and UTR7 belong to the LI lineage, while UTR1a, UTR1b, and UTR1c originate from the LII lineage (Chen and Gyllensten, 2014). Additionally, the 3′UTR RNAs have different conformational ensembles and shift folding dynamics. For instance, UTR1a, UTR1b/c, UTR2a/b, UTR2c, UTR4, and UTR7 have a ΔG of folding at 37°C of −111.40, −47.80, −52.20, −96.30, −48.60, and −48.0 kcal/mol, respectively (Supplementary Table S2). This indicates that the presence of SNPs changes the thermodynamics of the secondary structure folding of 3′UTR RNAs, which may subsequently shift miRNA binding sites.

### 3.2 Impact of SNPs within the MICA 3′UTRs on the gain and loss of binding of miRNAs

In our analysis, we examined the potential impact of SNPs within *MICA* 3′UTR on miRNA binding. Using the bioinformatic tool miRNASNP, we identified that the presence of SNPs can result in the gain or loss of binding sites for specific miRNAs, as outlined in Table 2. For instance, the presence of rs9266826 (G/A) generates binding sites to 6 miRNAs with a favorable ΔG value, such as hsa-miR-22-3p (−17.91 kcal/mol) and hsa-miR-4267 (−18.48 kcal/mol); conversely, 5 miRNAs, including hsa-miR-6078 (−21.18 kcal/mol), loose their binding sites to MICA 3′UTR in the presence of the same SNP. On the other hand, the presence of SNP rs9266829 (A/G) does not confer any miRNA binding gain but generates the loss of binding of hsa-miR-636 (−14.36 kcal/mol), hsa-miR-758-3p (−11.31 kcal/mol), and hsa-miR-581 (−13.83 kcal/mol). These findings acquire relevance when considering the dysregulation of miRNAs in cancer, as gaining or losing their binding to MICA transcripts could potentially affect the expression of the protein.

**TABLE 2:** Gain and loss of binding of miRNAs for presence of SNPs in the MICA 3′UTRs.

SNP in MICA 3′UTR	Gain	ΔG (Kcal/mol)	Loss	ΔG (Kcal/mol)
rs9266826 (G/A)	hsa-miR-6882-3p	−14.12	hsa-miR-6078	−21.18
	hsa-miR-1207-3p	−13.73	hsa-miR-4632-3p	−18.02
	hsa-miR-6514-3p	−12.37	hsa-miR-6746-3p	−20.22
	hsa-miR-4267	−18.48	hsa-miR-4485-5p	−16.24
	hsa-miR-22-3p	−17.91	hsa-miR-6849-3p	−14.44
	hsa-miR-6894-3p	−11.42		
rs9266827 (A/G)	hsa-miR-4733-5p	−13.46	hsa-miR-6720-5p	−18.97
	hsa-miR-7977	−18.26	hsa-miR-6512-3p	−19.11
			hsa-miR-3180-5p	−16.86
rs9266828 (G/C)	hsa-miR-183-3p	−4.58	hsa-miR-4760-3p	−4.63
	hsa-miR-4452	−8.64	hsa-miR-4272	−8.87
	hsa-miR-219a-2-3p	−10.81		
	hsa-miR-219b-3p	−7.37		
rs9266829 (A/G)			hsa-miR-636	−14.36
			hsa-miR-758-3p	−11.31
			hsa-miR-581	−13.83
rs140390705 (C/T)	hsa-miR-3910	−13.79	hsa-miR-548g-3p	−6.43
	hsa-miR-3714	−10.43	hsa-miR-4649-3p	−15.03
	hsa-miR-3129-3p	−5.21	hsa-miR-7162-3p	−12.40
	hsa-miR-4521	−9.46	hsa-miR-548av-3p	−8.58
	hsa-miR-506-3p	−16.06	hsa-miR-1827	−14.55
	hsa-miR-124-3p	−14.04	hsa-miR-6765-5p	−28.74
	hsa-miR-5583-5p	−7.78		
	hsa-miR-7844-5p	−10.88		
rs 1880 (C/A/T)	hsa-miR-520e-5p	−9.81	hsa-miR-147b-5p	−10.97
	hsa-miR-433-3p	−14.32	hsa-miR-12124	−10.86
	hsa-miR-7154-5p	−15.42	hsa-miR-10393-3p	−11.63
	hsa-miR-8060	−21.46	hsa-miR-4261	−16.97
			hsa-miR-4496	−24.78
			hsa-miR-6801-5p	−18.48
			hsa-miR-873-5p	−14.63
rs113015830 (T/A)	hsa-miR-1283	−12.80	hsa-miR-3529-3p	−9.95
	hsa-miR-606	−7.39	hsa-miR-3065-5p	−9.72
			hsa-miR-196a-1-3p	−10.87
rs1131904 (A/G)	hsa-miR-614 hsa-miR-1281	−14.22	hsa-miR-365b-3p	−9.28
		−13.42	hsa-miR-7853-5p	−10.93
			hsa-miR-105-5p	−11.76
			hsa-miR-532-5p	−12.39
			hsa-miR-365a-3p	−9.28
			hsa-miR-6836-3p	−11.63
rs9266831 (G/A)	hsa-miR-584-5p	−11.10	hsa-miR-183-5p	−13.87
	hsa-miR-5579-5p	−12.16	hsa-miR-8074	−14.28
	hsa-miR-150-3p	−13.72		
	hsa-miR-1263	−11.40		
rs9266825 (C/A)	hsa-mIR-7161-3p	−7.74	hsa-miR-335-5p	−11.78
	hsa-miR-3156-5p	−17.47	hsa-miR-558	−10.45
	hsa-miR-11181-5p	−9.25	hsa-miR-4487	−19.76
	hsa-miR-3200-5p	−11.51	hsa-miR-3160-3p	−17.31
	hsa-miR-4256	−12.37		
rs 1882 (A/G)	hsa-miR-645	−11.60	hsa-miR-874-3p	−14.1
	hsa-miR-6789-3p	−21.17	hsa-miR-3085-5p	−24.33
	hsa-miR-1249-3p	−13.75	hsa-miR-3157-3p	−16.68
			hsa-miR-6802-5p	−13.20
			hsa-miR-6800-5p	−18.48

### 3.3 Prediction of main putative miRNAs targets on different types of MICA 3′UTRs

We considered different types of *MICA* 3′UTR to predict potential miRNA targets with a high probability of binding to MICA 3′UTR. These included the specific combinations of SNPs described on Table 1 and Figure 3. Our analysis identified 12 miRNAs with potential binding sites to *MICA* 3′UTR variants, which exhibited diverse strength of interaction (ΔG ranged from −8.00 to −18.14 kcal/mol). The miRNA that meet these characteristics are hsa-miR-1184, hsa-miR-711, hsa-miR-335-5p, hsa-miR-1207-5p, hsa-miR-20b-5p, hsa-miR-93, hsa-miR-106a-5p, hsa-miR-17-5p, hsa-miR-20a-5p, hsa-miR-520d-3p, hsa-miR-106b, and hsa-miR-7156-3p, according to the nt position to which they bind (Supplementary Table S1). These miRNA fulfilled our predefined criteria, as they bind to their target seed sequences from canonical binding sites with Gibbs free energy values lower than −8.00 kcal/mol. Notable, some of these miRNAs, such as hsa-miR-1207-5p, hsa-miR-17-5p, hsa-miR-106a-5p, hsa-miR-106b, hsa-miR-20a-5p, and hsa-miR-93, showed multiple binding sites on MICA 3′UTR bearing SNPs, thus increasing the possibility of post-transcriptional regulation of MICA. Figure 3 shows a compilation of the DNA sequences encoding the 3′UTR for multiple *MICA* alleles, highlighting the presence of nine SNPs of interest within this region, along with the corresponding sequences of miRNAs and their seed sequences found in this region. We observed that hsa-miR-1184 exhibited a preferential binding site to the 3′UTR of MICA*008 (MFE = −13.9 kcal/mol due to the presence of a premature codon stop. However, other *MICA* alleles did not show the same binding capacity, since this site corresponds to their coding sequences. Additionally, a binding site for hsa-miR-711 found on the 3′UTRs of MICA*015 and MICA*017 (UTR2c), which could be affected by the presence of the rs9266825 SNPs, did not affect the 3′UTRs of other *MICA* alleles. These findings highlight the binding of miRNAs to specific *MICA* alleles, as different alleles exhibit distinct sequences.

**FIGURE 4:**
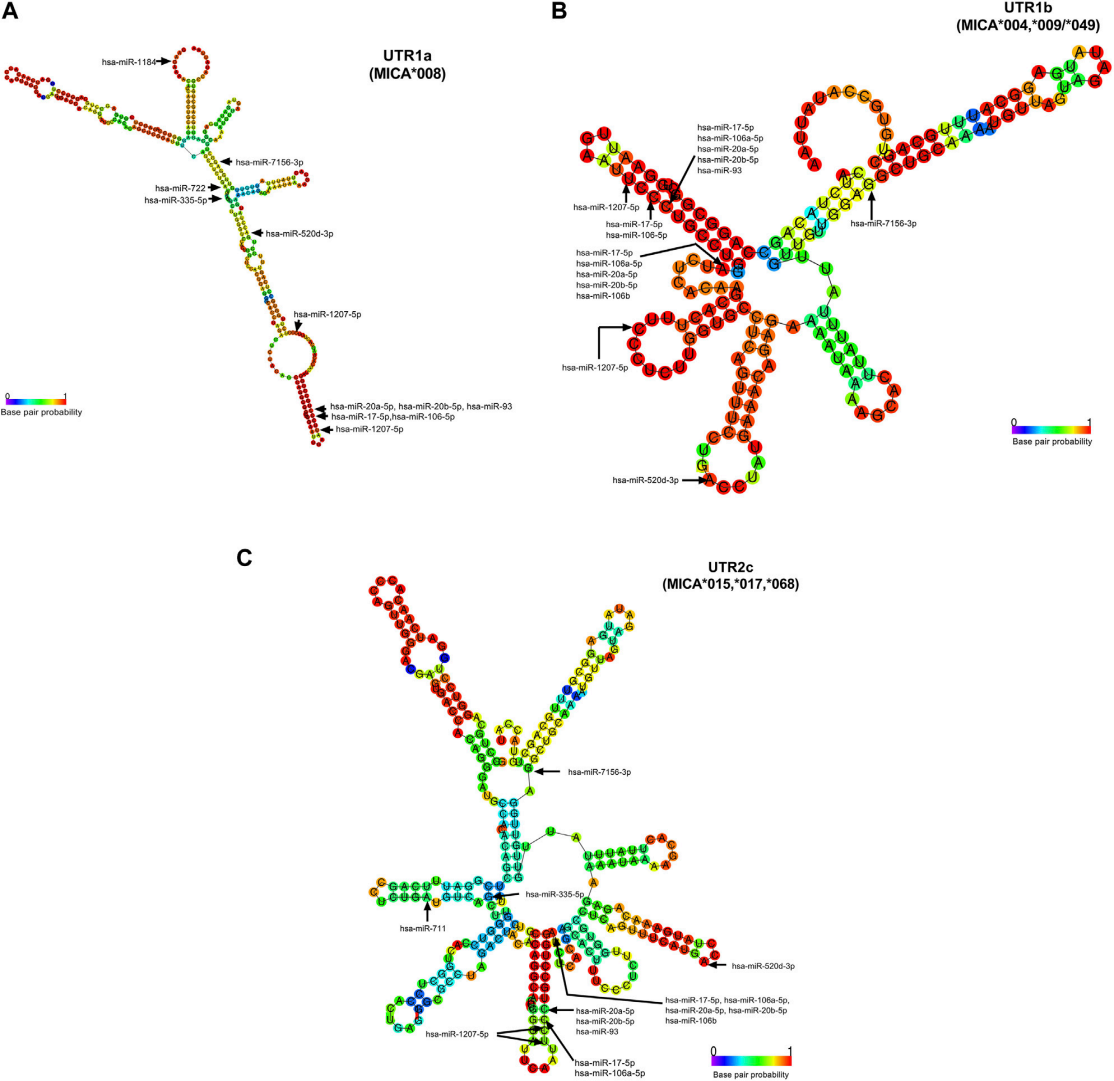
Accessibility of miRNAs to 3′UTR RNA fold of UTR1a (MICA*008), UTR1b (MICA*004, *009, *049), and UTR2c (MICA*015, *017, *). The base pair probability of 3′UTR RNA folding is indicated in the colored scale, where a lesser probability is highlighted in blue and a greater one in red. These structures presented folding capacity with the most favorable thermodynamic energy (Supplementary Table S2). The start nucleotide involved in the interaction of miRNAs with each UTR is indicated with arrows. **(A)** The folding structure of UTR1a is the largest and in consequence the folding is different to other 3′UTR types. The start nucleotide that binds to hsa-miR-1184, hsa-miR- 1207-5p, and hsa-miR-335-5p with UTR1a show that this latter has an unpaired nucleotide in its RNA folding structure, indicating the greater accessibility of these miRNAs to RNA. **(B)** The folding of UTR1b is similar to the UTR2a, UTR2b, UTR4, and UTR7 (not shown). This structure presents unpaired nucleotides that favor the binding of hsa-miR-1207-5p, hsa-miR-520d-3p, and a group of miRNAs (hsa-miR-17-5p, hsa-miR-106a-5p, hsa-miR-20a-5p, hsa-miR-20b-5p, and hsa-miR-106b). **(C)** The UTR2c folding has few nucleotides with a high probability of base pairs; however, this structure was the best prediction given by RNAfold for this sequence. The hsa-miR-520d-3p may be the only one with accessibility to this structure, since the start nucleotide involved in the binding is unpaired.

The accessibility of these miRNAs to 3′UTR sequences depends on the unpaired or paired nts involved in the binding in the seed sequence, which are generated from secondary RNA structures of each 3′UTR type, as depicted in Figure 4. For example, UTR1a and UTR1b, as well as UTR2, UTR4, and UTR7 exhibit different RNA folding patterns, which determines the availability of binding sites to miRNA. In the case of hsa-miR-1207-5p, this miRNA could access both, 3′UTR1a and 3′UTR1b, since the nt in position 2 in its seed sequence is unpaired. Conversely, hsa-520d-3p could access 3′UTR1b but not 3′UTR1a, whereas hsa-miR-335-5p showed the opposite pattern of accessibility.

### 3.4 The presence of SNPs in MICA 3′UTR within the binding site of miRNAs has a direct impact on their interaction strength

The presence of SNPs within the 3′UTR region can disrupt the binding site for certain miRNAs, which affects the strength of their interaction. Among the analyzed SNPs, rs9266829 (A/G) showed the highest potential as a target for miR-106a-5p, miR-106b, miR-17-5p, miR-20a-5p, miR-20b-5p, and miR-93, as shown in Table 3. Furthermore, the strength of interaction between these miRNAs and their respective target sites exhibited allele specificity, with UTR1a and UTR2a presenting higher ΔMFE values, ranging from 1 to 3 (Table 3). The difference in the binding energy between miR-20b-5p and UTR1a or UTR7 is the highest (ΔMFE = 2.9). Notably, miR-106b displayed potential binding exclusively to UTR1 (rs9266829A) in all its variations, including UTR1a, UTR1b, and UTR1c, while the presence of rs9266829G resulted in the loss of binding site for this miRNA. Intriguingly, the secondary structure of these *MICA* 3′UTRs revealed an unpaired nucleotide in the sequence involved in hsa-miR-106b binding (Figure 4), suggesting enhanced accessibility of the miRNA to its targets.

**TABLE 3:** Impact of SNPs on MICA alleles on miRNA interaction.

3′UTR SNP ID	Allele change	miRNA with SNP target binding	MICA UTR comparison	∆ MFE
**rs9266829**	A/G	hsa-miR-106a-5p	UTR1a vs. UTR7	1.87
		miR-17-5p	UTR1a vs. UTR7	1.87
		miR-20a-5p	UTR1a vs. UTR7	2
		miR-20b-5p	UTR4 vs. UTR1a/b/c	3.2
		miR-20b-5p	UTR7/UTR2a/b/c vs. UTR1a	2.9
		miR-93	UTR1 vs. UTR7/UTR2a/b/c	1.45–1.52
**rs1880**	C/A	miR-1207-5p	UTR7 vs. UTR2	2.04
**rs9266825**	C/A	miR-711	UTR1a vs. UTR2c	1.71

The binding site of hsa-miR-711, located within the rs9266825 variant, displayed binding energy only with the UTR1a and UTR2c alleles, resulting in MFE = −18.14 and −16.44, respectively). Although certain miRNA binding sites did not contain any SNPs, the evaluation of their interaction strength with different *MICA* 3′UTR regions indicated that variations in the binding energy could impact miRNA binding capacity.

### 3.5 SNPs outside the binding site of miRNAs indirectly influence their interaction strength with MICA 3′UTR

We identified an indirect influence of SNPs in the binding energy of hsa-miR-520d-3p, which exhibits variability depending on the type of 3′UTR. Even though hsa-miR-520d-3p does not bind to UTR1a, the accessibility of this miRNA to the secondary structure of the majority of the 3′UTRs is favorable due to the presence of unpaired nucleotides in the 3′UTR sequence, such as in the case of UTR1b, UTR2c, UTR4 and UTR7. Nevertheless, in the UTR1a, the nucleotide involved in the binding is already paired, thus affecting the binding potential of this miRNA. This observation suggests that the presence of SNPs harbored in the 3′UTR sequences could indirectly influence the binding sites of specific miRNAs due to changes in their RNA folding.

## 4 Discussion

The 3′UTR regions of *MICA* alleles display considerable diversity, resulting in variations in the binding energy to their potential miRNA. Consequently, these differences in binding probability can lead to diverse regulatory effects on MICA expression, particularly in the context of miRNA dysregulation in cancer and inflammatory processes. Notably, SNPs located within putative miRNA binding sites in the *MICA* 3′UTR have been shown to exert significant regulatory influence by affecting miRNA binding, either enhancing or reducing this phenomenon. In our study, we investigated the collective impact of SNPs within the *MICA* 3′UTR region, specifically focusing on the distinct 3′UTR types observed across different *MICA* alleles. Through bioinformatic approaches, we aimed to improve the prediction of miRNAs binding potential that may exert functional effects, thereby enhancing our understanding of MICA protein regulation.

The presence of SNPs in the 3′UTR region on genes involved in cancer has revealed that disruptions of the regulatory role of miRNAs can contribute to the development of cancer and autoimmune diseases (Yu et al., 2020; Tchacrome et al., 2022) SNPs in this region have been proposed as biomarkers of prognosis, prevention (Jeon et al., 2021), susceptibility and chemotherapy response (Cipollini et al., 2014), or even associated with cancer risk (Yu et al., 2020). The presence of multiple SNPs within this region explains, in part, the observed variability in cancer-related gene expression among patients. For instance, in non-small cell lung cancer (NSCLC), the rs9266825 and rs1882 *MICA* variants, which were included in our bioinformatic analysis, have shown a significant association with patient overall survival, suggesting these alleles a potential prognostic marker for NSCLC clinical outcomes (Xu et al., 2014). Additionally, MICA alleles have been associated with higher susceptibility to inflammatory diseases, mainly autoimmune diseases, such as ankylosing spondylitis (MICA *007) (Zhou et al., 2014), Behçet’s syndrome (MICA*009 and/or *019) (Hughes et al., 2005; Munoz-Saa et al., 2006), ulcerative colitis (MICA *007), and inflammatory bowel disease with type 2 peripheral arthropathy (MICA*008) (Orchard et al., 2001). As a whole MICA alleles may contribute to the pathophysiology and the different of miRNAs binding sites offered by these multiple alleles may impact on MICA regulation.

Hence, the presence of SNPs within MICA 3′UTR may prevent or generate binding sites to miRNA, resulting in the gain or loss of miRNA regulators of *MICA* gene expression impacting on cellular performance in the tumoral microenvironment, induction of damage, or tissue rejection. In the case of gaining or improved interaction strength to a miRNA binding site, these SNPs may promote MICA transcript degradation, resulting in reduced expression of the protein on the cell membrane. In cancer, reduced MICA expression diminishes the recognition of NK cells via the NKG2D activating receptor, thereby avoiding the antitumoral immune response (Xing and Ferrari de Andrade, 2020). As an example of this possible outcome, our analysis showed that UTR4 have increased ΔG *versus* UTR1a/b/c for miR-20b-5p (Table 3)**,** a miRNA that is upregulated in hepatocellular cancer (Zhang et al., 2014), suggesting that the presence of UTR4 could enhance down-expression of MICA by miR-20b-5p in this type of cancer; however, function assays are necessary to elucidate this MICA regulatory mechanism. On the other hand, the loss of binding sites for miRNAs may disrupt the control exerted by particular miRNA on MICA transcription. This disruption could lead to enhanced MICA expression, consequently inducing the activation of NK cells via the NKG2D receptor. This scenario has been found in patients with autoimmune disease, such as systemic lupus erythematosus (Spada et al., 2015); the magnitude of this response may rely on the specific MICA variant, which may possibly result in increased tissue damage.

In the tumor environment, certain MICA variants that express methionine in the 129-amino acid (MICA-129 Met), such as the allelic variants *001, *002, *007, *011, *012, *015, *017, and *018 have been reported to exhibit a higher affinity for NKG2D receptor and potentially lead to a strong cytotoxic response (Isernhagen et al., 2016). However, MICA expression should be viewed with caution, because of cleavage susceptibility by metalloproteases expressed by tumor cells (Waldhauer et al., 2008; Chitadze et al., 2013). This cleavage results in the release of a soluble form of MICA, which has the capacity to downregulate the NKG2D receptor, thereby reducing the cytotoxic response of NK cells (Groh et al., 2002). Furthermore, upon translation, MICA*008 variant is also released via exosomes, which exhibits a potent downregulation effect on the NKG2D receptor (Ashiru et al., 2013). Consequently, the pathological effects of miRNAs depend on several factors, including MICA3′UTR alleles, miRNA expression profile within each cell type, and the stage of tumor development.

To provide a comprehensive assessment of the SNPs present in each type of MICA 3′UTR on the potential impact on miRNA binding, we grouped all *MICA* variants described to date into clusters to redefine the types of 3′UTR. This novel classification not only enhances our understanding of their features but also enables the application of our methodology to identify potential targets for miRNA binding to other genes. It is interesting to note the different thermodynamics of the secondary structure of the 3′UTRs, whose characteristics may be related to the presence of SNPs and to the length of their sequences. These changes in the secondary structure and the thermodynamic stability of 3ÚTR could implicate functional consequences, such as changes in protein expression (Chen et al., 2008; Ceolin et al., 2016).

Alternative polyadenylation and alternative splicing are mechanisms that contribute to the 3′UTR diversity, promoting extension or shortening of this region, and influence mRNA regulation by gain or loss of miRNAs binding sites (Han et al., 2018). These alternate 3′UTR isoforms can impact transcriptional regulation, affecting stability, translation and RNA localization (Tian and Manley, 2013). Although our study did not analyze alternative polyadenylation patterns in MICA alleles, future analysis should take these patterns into account, particularly considering the type of cell, as they are often tissue-specific and have potential to influence on biological cellular processes (MacDonald, 2019; Arora et al., 2022).

Herein we propose new and specific miRNAs with a high probability of binding to different 3′ UTR types on *MICA* alleles applying free-access bioinformatic tools. We identified differential binding sites and binding energies between miRNA and 3′UTR types. We found that hsa-miR-1184 and hsa-miR-335-5p may regulate mainly UTR1a, while hsa-miR-711 may regulate UTR1a and UTR2c; in turn, hsa-miR-106b may regulate all subtypes of UTR1. Also, hsa-miR-1207-5p, hsa-miR-20b-5p, hsa-miR-17-5p, hsa-miR-520d-3p, and hsa-miR-7156-3p showed binding potential to different types of UTRs depending on the energy binding strength. It would be interesting to perform *in vitro* studies using these miRNAs to analyze their effect on MICA expression, especially in cancer, as MICA plays a crucial role in immune surveillance against this disease. Additionally, we also have identified several miRNAs (hsa-miR-20a-5p, hsa-miR-93, hsa-miR-106a-5p, and hsa-mir-106b) that have been previously reported to be upregulated and act as down-regulators of MICA expression in various types of cancer (Stern-Ginossar et al., 2008; Kishikawa et al., 2013; Wu et al., 2014; Xie et al., 2014; Yang et al., 2015; Jafarzadeh-Samani et al., 2017; Shen et al., 2017; Tang et al., 2019; Shekari et al., 2020; Awad et al., 2021), which supports our prediction approach.

Although the binding site of hsa-miR-1184 is not polymorphic, this miRNA may regulate the expression of MICA in patients carrying UTR1a, whereas its hybridization could be less efficient in other UTRs, since its binding region corresponds to its coding sequences. In addition, hsa-miR-1184 plays a significant role in various types of cancers and its impact on MICA expression should be addressed. In colorectal cancer, the overexpression of this miRNA inhibits cell proliferation and promotes apoptosis by circular RNA hsa_circ_0128,846 (Chen S. et al., 2020), whereas in triple-negative breast cancer (TNBC) subtype tissue and cell lines it is linked to enhanced proliferation, migration and invasion by circular RNA lar hsa_circ_0000732 (Chen et al., 2022). Furthermore, the downregulation of this miRNA promotes tumorigenesis (Wang et al., 2020a) in colorectal cancer through IGFBP2 and ITGA3 genes, and in bladder cancer, it is associated with accelerated tumor progression (Jiang P. et al., 2020; Yang et al., 2020), hsa-miR-1184 has been proposed as a potential serum biomarker to distinguish between benign prostatic hyperplasia (BPH) and prostate cancer (Knyazev et al., 2016). Thus, hsa-miR-1184 is an interesting target for further studies on its regulatory role in cancer-related processes.

Hsa-miR-711, which may regulate MICA expression on patients carrying UTR1a and UTR2c, has been associated with osteosarcoma-associated proliferation, migration, and invasion; it is regulated by circular RNA circ_0008792 (Chen L. et al., 2020). Conversely, in gastric cancer, the expression of hsa-miR-711 is low and its overexpression downregulates adhesion molecules, inhibiting the epithelial-mesenchymal transition (EMT) (Xiao et al., 2018). Similarly, this miRNA has been found in extracellular vesicles (EV), which inhibits the expression of CD44 in bone marrow mesenchymal stem cells in chronic myelogenous leukemia (Jiang Y-H. et al., 2020). The presence of miRNAs in extracellular vesicles suggest that MICA expression on tumor cells can be regulated by miRNA carried into EVs.

Herein we also highlight hsa-miR-1207-5 miRNA, which displayed binding sites for different 3′UTR: two binding sites for UTR1a UTR1b, and UTR1c and one binding site for other *MICA* UTRs. In both cases, this miRNA could have a favorable accessibility to the secondary structure of 3′UTR. Also, hsa-miR-1207-5p could bind with high strength to UTR1 and UTR4; in turn, it may bind to UTR2 and UTR7 in another region with lower interaction force, thus giving this miRNA a versatile effect depending on the genetic component and the tumor microenvironment. The involvement of hsa-miR-1207-5p in various cancer-related processes has been extensively documented. The evidence shows it is associated with tumor suppressive activity in several types of cancer (Wang and Wu, 2017; Cui et al., 2019; Yan et al., 2020); it has also been regarded as a negative regulator of the expression of several genes involved in epithelial-to-mesenchymal transition (EMT) (Dang et al., 2016; Qin et al., 2016). In colorectal cancer, this miRNA suppresses cell proliferation, migration and invasion through targeting FMNL2, a downstream molecule of a circular RNA related as an oncogene in this type of cancer (Yan et al., 2020); its downregulation has also been associated with shorter overall survival rate in patients (Wang and Wu, 2017). Recently, hsa-miR-1207-5p has been proposed as a biomarker for diagnosis and prognosis of colorectal cancer (Wang et al., 2020b). In lung cancer, it participates in macrophage function control and facilitates the release of proinflammatory chemokines throughout its target colony stimulating factor 1 (CDF1); it can also inhibit STAT3 and AKT signaling (Dang et al., 2016). In gastric cancer, hsa-miR-1207-5p exerts its effects on telomerase reverse transcriptase mRNA (Chen et al., 2014), and promotes proliferation by a long noncoding RNA BC032469 (Lu et al., 2016). In nasopharyngeal carcinoma, hsa-miR-1207 has been shown to be inhibited by non-coding RNA Pvt1 or long non-coding RNA 319, which may act as a sponge to miRNAs, inducing cellular proliferation via PI3K/AKT (Xiao et al., 2018), or carcinogenesis via KLF12 signaling pathways (Nunziato et al., 2019), respectively. In prostate cancer, hsa-miR-1207-5p is downregulated and associates with the androgen receptor via FNDC1, a protein that contains a conserved protein domain of fibronectin and it is upregulated in this type of cancer (Das et al., 2016). In contrast, in breast cancer, this miRNA is upregulated and promotes tumor cell proliferation through STAT6 regulation (Yan et al., 2017). Taken together, these findings suggest the importance of uncovering the targets of miRNA, as well as deciphering their regulatory potential over gene expression, including *MICA*. The identification of the expression levels of miRNAs in each type of cancer, the presence of SNPs in miRNAs (Ryan et al., 2010), and their effect on MICA expression could give us a major comprehension of their biological effects in this context. Such molecular features are worth exploring using *in vitro* studies in order to move beyond the limitations of bioinformatic tools alone (Rojo Arias and Busskamp, 2019).

The effect of regulation of gene expression by miRNAs binding to 3′UTR has been widely studied, so has their regulation through coding sequences (CDS), although with less pronounced effects (Friedrich et al., 2020). This is in agreement with a number of reports analyzing CDS-located miRNA binding sites and may be due to the accessibility of the target site in the context of the translation process (Gu et al., 2009). In this work, we focused on studying solely the regulation of *MICA* 3′UTR by miRNA targets; however, it will be valuable to use bioinformatic tools that include the coding sequences of the genes (Liu et al., 2013). The integration of CDS into bioinformatic tools may improve the efficiency and accuracy of binding prediction relative to algorithm trained with 3′UTR information (Bertolazzi et al., 2020; Lin et al., 2023).

While our initial bioinformatics analysis focused on elucidating the potential interactions between miRNAs and MICA 3′UTR allelic sequences, we acknowledge that this computational approach does not directly unveil the concrete impacts of the selected miRNAs. In this sense, we recognize the imperative need to bridge this gap and carry out experimental approaches to substantiate and delineate the actual effects of miRNAs in the context of our work.

In conclusion, *MICA* post-transcriptional regulation by miRNAs should be carefully studied, considering the diversity of *MICA* 3′UTR types within specific *MICA* alleles. The presence of SNPs in this region could promote gain or loss of binding sites or, alternatively, significantly impact the binding energy; enhancement or detriment of binding may also have a huge importance in the context of cancer and other diseases. Our *in silico* approach sets the stage for future investigations in cancer and inflammatory models, where empirical experiments will be pivotal to establish conclusive insights, thus advancing our understanding of the intricate relationship between miRNAs and MICA allelic variability.

## Data Availability

The raw data supporting the conclusion of this article will be made available by the authors, without undue reservation.
