# Supplementary material for: Impact of MICA 3′UTR allelic variability on miRNA binding prediction, a bioinformatic approach
Source: Front Genet. 2023 Dec 7;14:1273296. doi: 10.3389/fgene.2023.1273296 (PMC10749337; doi:10.3389/fgene.2023.1273296)
Supplement: Supplementary file 1 [file Table1.pdf]

Supplementary Material

Supplementary Figures

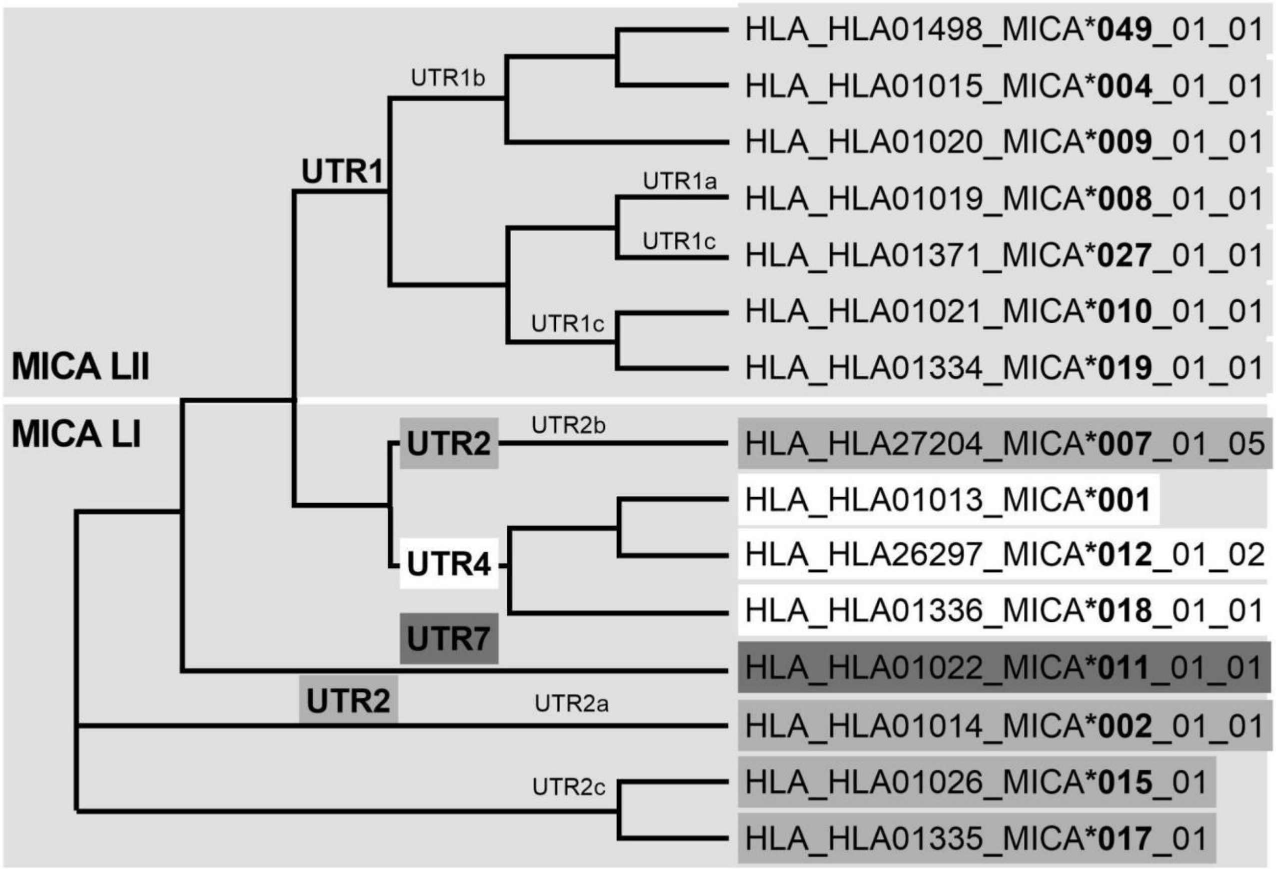

Figure S1. Possible polyphyletic origin of MICA 3'UTRs. The 3'UTR of *MICA* alleles analyzed in this study bifurcate in two lineages, LI and LII. This phylogenetic tree was constructed using *MICA* allele sequences reported in the IPD-IMGT/HLA Database. The phylogenetic tree is based on the similarity of the sequences aligned in the Clustal Omega EMBL-EBI Webserver. For intance, *MICA* alleles with UTR1 (including UTR1a, UTR1b and UTR1c) belong to LII lineage, whereas alleles with UTR2, UTR4 and UTR7 belong to LI lineage.

Supplementary Tables

Table S1. Binding energy of miRNAs of interest with target in MICA 3'UTR.

Hsa-miR	Nucleotide position Start	Nucleotide position End	UTR1 a *008	UTR1 b *004 *009 *049	UTR1 c *010 *019 *027	UTR2 a *002	UTR2 b *007	UTR2 c *015 *017	UTR4 *001 *012 *018	UTR7 *011
**1184**	1003	1019	-13.99	–	–	–	–	–	–	–
**711**	1115	1132	-18.14	–	–	–	–	-16.43	–	–
**335-5p**	1121	1127	-9.32	–	–	–	–	NC	–	–
**1207-5p**	1213	1239	-12.78	-13.1	-13.1	--	--	--	-12.55	-13.92
	1186	1193	--	--	--	-11.88	-11.88	-11.88	--	--
**20b-5p**	1189	1212	--	--	--	-11.99	-11.99	-11.99	-12.2	-11.92
			--	-8.95	-8.95	--	--	--	--	--
	1197	1212	-9.02	--	--	--	--	--	--	--
**93**	1189	1212	--	--	--	-10.83	-10.83	-10.83	-11.04	-10.46
			-9.31	-9.29	-9.29	--	--	--	--	--
**106a-5p**	1197	1212	-10.8	-10.63	-10.63	--	--	--	--	--
	1188	1212	--	--	--	-9.32	-9.32	-9.32	-9.5	-8.93
**17-5p**	1197	1212	-10.7	-10.53	-10.53	--	--	--	--	--

	1188	1212	--	--	--	-9.22	-9.22	-9.22	-9.4	-8.83
**20a-5p**	1197	1212	-10.62	-10.45	-10.45	--	--	--	--	--
	1189	1212	--	--	--	-8.69	-8.69	-8.69	-8.9	-8.62
**520d-3p**	1237	1266	-10.25	-10.61	-10.61	-8.93	-7.93	NC	-10.26	-9.97
**106b**	1197	1212	-9.49	-9.31	-9.31	NC	NC	NC	NC	NC
**7156-3p**	1283	1289	NC	NC	-8.63	-8.88	-8.88	-8.88	-8.7	-8.76

Energy is expressed as kJ mol-1. NC: Does not meet criteria.

Tabla S2: Thermodynamic parameters of RNA folding of the MICA 3'UTR types according to UNAfold and RNAfold analysis

MICA 3'UTR type	ΔG RNA fold (Kcal/mol) by UNAfold	ΔG RNA fold (Kcal/mol) by RNAfold
UTR1a	-111.40	-114.66
UTR1b	-47.80	-52.17
UTR1c	-47.80	-52.17
UTR2a	-52.20	-56.84
UTR2b	-52.20	-56.84
UTR2c	-96.30	-98.81
UTR4	-48.60	-53.06

UTR7	-48.10	-52.53
